# Gastrointestinal Tract Polyp Anomaly Segmentation on Colonoscopy Images Using Graft-U-Net

**DOI:** 10.3390/jpm12091459

**Published:** 2022-09-06

**Authors:** Muhammad Ramzan, Mudassar Raza, Muhammad Imran Sharif, Seifedine Kadry

**Affiliations:** 1 Department of Computer Science, COMSATS University Islamabad, Wah Campus, Islamabad 47040, Pakistan; 2 Department of Applied Data Science, Noroff University College, 4612 Kristiansand, Norway; 3 Department of Electrical and Computer Engineering, Lebanese American University, Byblos 999095, Lebanon

**Keywords:** segmentation, convolutional neural network, deep learning, gastrointestinal tract, health informatics

## Abstract

Computer-aided polyp segmentation is a crucial task that supports gastroenterologists in examining and resecting anomalous tissue in the gastrointestinal tract. The disease polyps grow mainly in the colorectal area of the gastrointestinal tract and in the mucous membrane, which has protrusions of micro-abnormal tissue that increase the risk of incurable diseases such as cancer. So, the early examination of polyps can decrease the chance of the polyps growing into cancer, such as adenomas, which can change into cancer. Deep learning-based diagnostic systems play a vital role in diagnosing diseases in the early stages. A deep learning method, Graft-U-Net, is proposed to segment polyps using colonoscopy frames. Graft-U-Net is a modified version of UNet, which comprises three stages, including the preprocessing, encoder, and decoder stages. The preprocessing technique is used to improve the contrast of the colonoscopy frames. Graft-U-Net comprises encoder and decoder blocks where the encoder analyzes features, while the decoder performs the features’ synthesizing processes. The Graft-U-Net model offers better segmentation results than existing deep learning models. The experiments were conducted using two open-access datasets, Kvasir-SEG and CVC-ClinicDB. The datasets were prepared from the large bowel of the gastrointestinal tract by performing a colonoscopy procedure. The anticipated model outperforms in terms of its mean Dice of 96.61% and mean Intersection over Union (mIoU) of 82.45% with the Kvasir-SEG dataset. Similarly, with the CVC-ClinicDB dataset, the method achieved a mean Dice of 89.95% and an mIoU of 81.38%.

## 1. Introduction

The stomach, small intestine, and large intestine (which include the colon, rectum, and anus) are the parts of the gastrointestinal tract (GI tract) [1,2]. The GI tract is the core part of the digestive system of the human body where mucosal findings vary from mild to extremely lethal diseases [3,4]. The mucous membrane has protrusions of abnormal tissue referred to as polyps. Polyps can grow in the GI tract in any place, but most are found in the colorectal area. Non-neoplastic and neoplastic are the two categories of colorectal polyps [5]. Non-neoplastic polyps can be divided into subcategories—hyper-plastic, hamartomata’s polyps, and inflammatory—which are recognized as non-cancerous diseases. On the other hand, neoplastic polyps can become cancerous depending upon the size of the polyps. The growth of polyps mostly takes place in the colorectal area (inner tissue lining); they are non-cancerous but indorse colorectal cancer (CRC), which is a very dangerous and lethal disease. The scope of CRC across the world accounts for nearly 10%, of all cancer-related deaths [6]. The colorectal polyps are analyzed and removed after examining the colon using a standardized colonoscopy procedure. There are different endoscopy methods to examine the GI tract, but Confocal Laser Endomicroscopy (CLE) is a cutting-edge and microscopic-level endoscopic technique that allows for subcellular imaging and optical biopsies to be performed while the patient is being examined. The Colonoscopy expert can use CLE to observe real-time histology images, as well as examine the GI tract, connective tissue, and mucosal cell structure [7]. Histopathology examination (HE) is manually performed by a gastroenterologist for polyp or tumor removal. Neoplastic lesions (adenomatous polyps) are resected to reduce CRC [8]. Similarly, the survival rate is increased by diagnosing colon cancer at its early stage. Endocytoscopy is used in the NBI mode, which allows the endoscopist to acquire real-time microvascular photos with a magnification of 520 [9]. The colonoscopy procedure depends on the operator, who can make mistakes that increase the chance of a higher miss rate of the adenomatous polyps. The size of the polyp can be on a macroscopic level in the tissue of the colon, which provides a hindrance to manual disease detection. Additionally, manually screening is a time-consuming task that requires the doctor to have experience and ability [10]. A procedural colonoscopy is a time-demanding, expensive, and aggressive process whereby air insufflation and a high-quality bowel are required during examination [11]. Colonoscopy data are collected in the form of videos by the clinical centers. The endoscopist captures data in tough routines that are not used efficiently for clinical diagnosis procedures [12]. The number of frames captured in video colonoscopy cannot be observed properly in real-time, which increases the miss rate. CADx systems are employed resourcefully for disease detection and the delineation of polyps. Computer vision and system design are successfully led in medical fields to develop accurate and efficient systems that mainly depend on well-organized data [13,14,15,16,17,18,19]. Similarly, there is a big bottleneck of public data available for accelerating the development of robust algorithms in this realm [20]. Automatic polyp segmentation has become a thought-provoking task because of the disparities in the shapes, positions, sizes, colors, appearances of polyps, and their masking with mucosa, stool, and other materials that are a hindrance in the correct diagnostics [21]. In the previous studies, different methods of feature extraction (feature map, patterns, color, etc.) were employed for polyp detection, semantic segmentation, localization, and classification [22,23]. Previous studies have found a high rate of missed detection. Recently, emerging convolutional neural network (CNN) deep learning methods have offered a solution to overcome the above-addressed challenges and also improve polyp detection accuracy during colonoscopy. Automatic polyp segmentation is crucial in the medical field. The computer-based identification and localization of polyps using frames of colonoscopy can save time for clinicians, and it also helps them to concentrate on more severe cases. A recent study revealed that deep learning-based automatic polyp segmentation has become a crucial research area that has achieved high accuracy using colonoscopy images and videos [24]. Preferably, a consistent, reliable, and robust computer-aided diagnostics (CADx) system is needed for polyp detection and segmentation.

In this manuscript, a CADx system is proposed for disease segmentation that comprises three phases: preprocessing, encoder, and decoder. In the preprocessing phase, the Contrast-Limited Adaptive Histogram Equalization (CLAHE) technique is performed for contrast improvement, which helps to extract more sophisticated features from the frames of the colonoscopy. A CNN-based deep learning model named Graft-U-Net is proposed, which consists of encoder and decoder blocks that explore and synthesize the features, respectively. The advised model is trained and evaluated using two publicly available datasets, CVC-ClinicDB and Kvasir-SEG. The results are matched to the existing results of UNet [25], ResUNet models [26], and ResUNet++ [27], which shows that the suggested method outperforms the existing methods. The contribution of this manuscript is summarized as follows:

- The CLAHE technique is applied at the preprocessing stage over the Kvasir-SEG dataset for improving the contrast of the frames, which has an impact on the overall execution of the deep learning model.
- A CNN-based 74-layer Graft-U-Net architecture is proposed, which is composed of an encoder (analyzing) and decoder (synthesizing) block. In the encoder and decoder blocks, different depth sizes of the filters are employed: 8,16,32,48, and 64. The encoder is modified by the inclusion of the grafting layers parallel to the conventional UNet layers in the encoder block. The derivations of the features of parallel networks are added and forwarded to the next layers. The results of the model are improved by including a graft network layer in the encoder block.

The organization of this document is as follows: The associated work is stated after the introduction in Section 2. In Section 3, the materials and methods for the proposed Graft-U-Net structure and polyp detection and segmentation are addressed. In Section 4, the results of the performed experiments and discussion are presented. In Section 5, the final remarks, consisting of a conclusion and discussion of future work, are summarized

## 2. Related Works

Automatic disease detection and segmentation have become active research areas in the past decade [28,29,30,31,32,33,34,35]. Several algorithms and efficient methods have been developed for polyp detection. With the development of methods and algorithms, the texture and color of the polyps were focused on in one research paper by applying handcrafted descriptors for learning features [36]. An existing study reveals that CNN has become a very famous method in the research industry for the accomplishment of public challenges in the computer vision field [37]. By using CNN, software modules and algorithms have been designed for edge and polyp detection in the frames [38]. Colonoscopy images and videos have been used for polyp detection via region-based CNN methods, including transfer learning (Inception and ResNet) and post-processing techniques [39]. The framework has been performed for disease detection and segmentation problems using the Generative Adversarial Network (GAN) model [40]. Real-time performance and high-sensitivity algorithms, including the YOLO algorithm, have been developed for polyp segmentation [41]. Transfer learning for polyp segmentation has been evaluated in terms of specificity and sensitivity [42]. The computer vision approaches have been improved due to the inclusion of data-driven methods for polyp segmentation [43]. Object segmentation has been performed using the down- and up-sampling techniques for the pixel-wise classification of polyps [44]. The fully convolutional network (FCN) has been suggested by Long et al. for polyp dissection [45].

UNet is the modified and extended architecture of the FCN [46]. Unet comprises an analysis path and a synthesis path that are recognized as an encoder and a decoder, respectively. The analysis part provides the detail of the deep features, while the synthesis part offers segmentation based on learned features. The encoder–decoder network is a very core component in terms of semantic segmentation in UNet and the FCN [30]. Multiple variants of UNet for biomedical segmentation are found in the literature. The encoder–decoder in UNet applies convolution layers whereby the encoder extracts essential semantic features ranging from down- to up-level. Table 1 depicts a summary of the existing models that are used for polyp segmentation using the Kvasir-SEG dataset.

The decoder generates the required segmentation mask by using extracted features from the encoder. The up-sampled (decoder) features are concatenated with the down-sampled (encoder) features using a skip connection. The final output binary masks are produced by the convolutional layers. The pre-trained network, including VGG16 and VGG19 [53], is replaced by the encoder stage of the UNet model for polyp segmentation tasks. The residual networks are very successful in transfer learning, such as ResNet50 for disease detection and localization [54]. Identity mapping and 3 × 3 convolutional layers are used by the residual network [55]. Vanishing gradients and exploding gradients are eliminated in a deeper neural network using identity mapping [56]. Several clinical endoscopy and colonoscopy image datasets are publicly available, and researchers can use them. In the proposed work, two datasets, CVC-ClinicDB and Kvasir-SEG, are employed for model evaluations.

## 3. Materials and Methods

**Figure 1:**
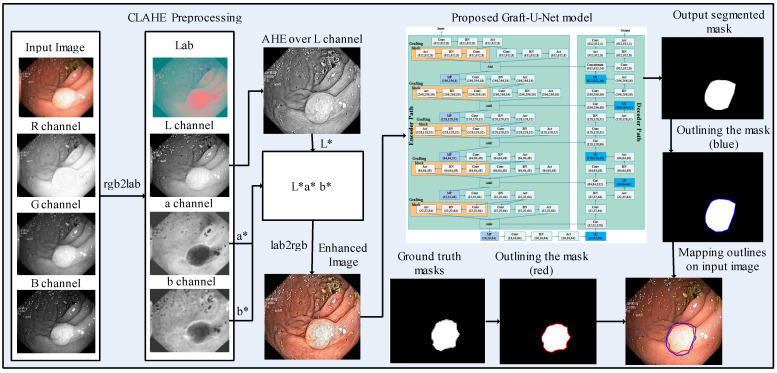
Block diagram of the proposed method for polyp segmentation.

A model, Graft-U-Net for polyp detection, is proposed, which comprises three main phases, including preprocessing, the encoder (analysis path), and the decoder (synthesis path). The CLAHE technique is used in the preprocessing stage, which enables the features to be more clearly visualized in the frames. The frames are given as an input to the encoder block, which explores the context of the frame without determining the location of the disease. The decoder follows the encoder for synthesizing the frames. The location is determined by using the skip connection initiated from the encoder block. The segmented mask and ground truth mask are outlined over the original frame with blue and red colors, respectively, for the analysis of the model. The block diagram of the method for polyp segmentation is demonstrated in Figure 1. A detailed description of each block is provided in the upcoming section.

### 3.1. Preprocessing

**Figure 2:**
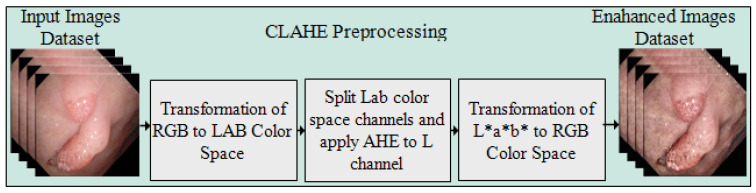
Flow diagram of CLAHE preprocessing method. (a) Original frames and (b) preprocessed frames.

Preprocessing is the first and most important stage of the presented approach for enhancing the intensity of pixels in the images. The CLAHE method is applied over the complete Kvasir-SEG dataset. The controlled intensity level of the pixels provides the local details in the image. The image is separated into corner areas, border, and inner regions, with the non-overlapping regions of equal size. The noise in the frames is clipped by setting the threshold of the clipper, which is not an easy task where the maximum redistribution level of the clipping and histogram levels are kept equal. The clip limit is defined by Reza [35], and the form of the equation is represented as below.

(1) $$\beta =\frac{M}{N}\left(1+\frac{\alpha }{100}\left(S_{\max}-1\right)\right)$$

where in each region of the image, M and N are the gray levels and resolution of the frame, respectively. α is a clipping factor with a range of [0−100] and $S_{max}$ shows the limited slope of the transformation function; thus, $\left[1-S_{max}\;\right]$ represents the slop range in each mapping. Figure 2 illustrates the preprocessed frames using the CLAHE method.

### 3.2. Proposed Graft-U-Net Model

**Figure 3:**
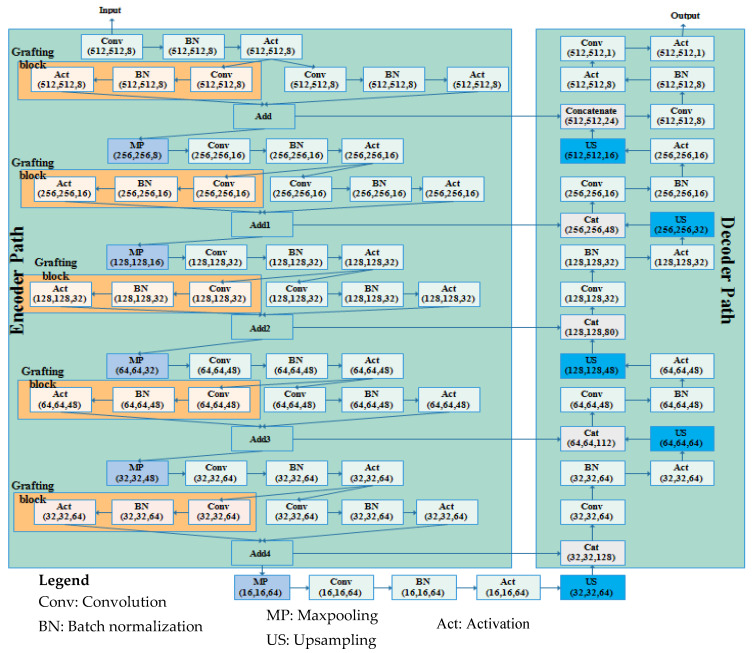
The architecture of the Graft-U-Net model.

Graft-U-Net is composed of encoder and decoder blocks whereby each encoder block includes the down-sample blocks (DSB). The five DSBs are created in the encoder block, passing feature maps one after another up to the fifth DSB. In every DSB, grafting blocks are proposed, parallel to the conventional layers in the encoder of UNet. Thus, the name Graft-U-Net is given to the network, which is a modified form of UNet. The decoder consists of five up-sampling blocks (USB) that are used for synthesizing the information using a skip connection. The architecture of Graft-U-Net’s composed encoder–decoder is depicted in Figure 3.

The USB receives the explored information from the DSB block and synthesizes the information to localize the disease location information by using a skip connection. The early information is determined by skip connections from the encoder to the decoder block. The whole set of USBs provides the disease location and also improves the model performance through advanced feature construction. A detailed explanation of each encoder (analysis) and decoder (synthesis) block is addressed in Section 3.2.1 and Section 3.2.2, respectively.

#### 3.2.1. Encoder DSB Blocks (Analysis Blocks)

The encoder block of the proposed Graft-U-Net consists of five DSBs. Each phase of the encoder block is distributed with two parallel networks, including the grafting layers network and a conventional network. Each network is created with different layers (convolution, batch normalization, and activation). The convolution layers provide a set of feature maps. Feature maps, after the activation of the layers of each network in every phase of the encoder block, are added and forwarded to the max-pooling layer. The sequence of the operation in the encoder block, with mathematical derivation, is defined as follows.

The size of the input frames is kept at 512 × 512 and provided to the network; then, the convolutional operation is performed with two input variables: three-channel color images, with the dimensions of the *n* and *c* channels being (*n × n × c*), and a 3D volume filter (Kernel) with a size of (*f × f × c*). The relationship between the input (images) and output (feature maps) is described below:

(2) $$G_{output}=\left\lfloor \frac{I_{size}-f_{size}+2p}{S}\right\rfloor +1$$

After the convolution operation, the batch normalization (BN) technique is implemented. After the feature normalization technique, BN is used to measure the variance and average in chunks for every feature. Additionally, channels of neurons are rationalized by setting the feature value of the small batches. The standard deviation is determined for splitting and extrapolating the average of the characteristics [57]. The average of the batch is represented mathematically as:

(3) $$Average_{Batch}=\frac{1}{N}\sum_{i=1}^{N}f_{i}$$

where $Batch=\{f_{1},f_{2}\ldots ,f_{i}\}b$, f is a feature of the batch set, and the variance of the small-batch is represented as:

(4) $$Variance_{Batch}=\frac{1}{N}\overset{N}{\underset{i}{\sum }}{\left(f_{i}-Average_{Batch}\right)}^{2}$$

Then, the features are normalized as:

(5) $$\hat{f_{i}}=\frac{f_{i}-Average_{Batch}}{\sqrt{Varaince_{Batch}+\sigma }}$$

where constant σ represents the steadiness of the features. The features are scaled between 0 and 1 using the activation function. The mathematical equation of ReLU is given as:

(6) $$ReLU_{out}=\max\left(0,x\right)$$

where *x* is the feature set of the frames. The complete set of features undergoes the application of convolution operation, BN, and the ReLU activation function, and is and passed to the next convolution layer network, which is represented by the equation below:

(7) ƛ=α(f(wx)+b)

where ƛ is the output feature set that is obtained across the graft layer network, α represents batch normalization, f is the activation function, and w and x represent the weight and the input feature maps to the convolutional layers, respectively. Bias is represented by *b*. The feature set of the first convolution layer network is forwarded to the graft layer network. The graft layer network is composed of the convolution layer, BN, and activation layer. The obtained information from the graft layer network is presented below:

(8) ϑ=β(f(wx)+b)

where ϑ is the output of the grafted convolution layer, β represents the batch normalization layer of the graft network, f is the activation function, w and x represent the weight and the input feature maps to the convolutional layers, respectively, and b determines the bias of the neuron. The collected information from the graft layer network is added to the parallel convolution layer network and is presented in the equation below:

(9) $$H(x)=\left[\sum_{i,j}^{N}{ƛ}_{i,j}+\sum_{i,j}^{N}\vartheta_{i,j}\right]$$

where H(x) is the obtained feature map after the addition of the feature maps of two networks, including the graft network and the parallel convolution layer network. After the inclusion of the layer, the feature set is passed to the max-pooling layer. The convolution operation decreases the resolution of the frames but increases the receptive field (context) information, which is covered by the filter at any given time. Channel-wise attention is given via the squeeze and excitation layers. It is formed of a two-step approach: a max-pooling operation squeezes the *n* number of feature vectors, and n shows the feature map count. In the upcoming step, the feed-forward network obtains the global feature vector from the squeeze net onward. After that, the features are reduced, and then, expanded to the original size *n*.

**Figure 4:**
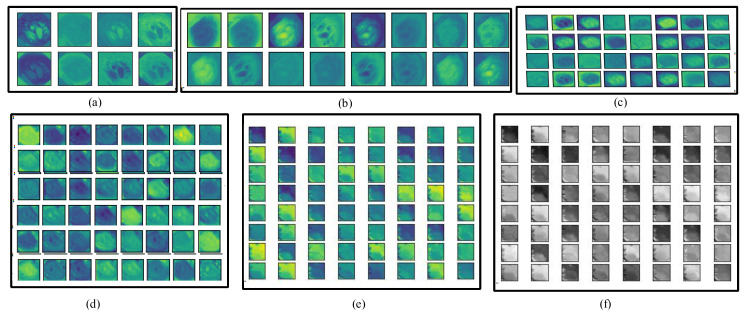
Visualization of various layers of Graft-U-Net. (**a**) C1, (**b**) C4, (c) C7, (**d**) C10, (**e**) C13, and (**f**) C13 (gray level).

In the whole encoder block, the convolution, BN, activation, and max-pooling operations are performed, and the depth of the frame is increased. In the convolution operation, different numbers of filters are employed (8, 16, 32, 48, and 64) with a filter size of 3 × 3. The number of filters is increased gradually from the upper to lower blocks, which helps to explore more detailed features in the frames. The information on polyp disease is analyzed, with in-depth analysis of the features. The encoder block provides overall context information, but not actual information on the location of the disease. For obtaining information on the location, a decoder block is required that uses the skip connection for collecting disease location information and increasing the resolution of the frames. Figure 4 depicts the visual information obtained from the different convolution layers (C1, C4, C7, C10, C13 in color, and C13 in grayscale) of the encoder block of Graft-U-Net using the kvasir-SEG dataset.

#### 3.2.2. Decoder USB Blocks (Synthesis blocks)

The decoder obtains the feature maps from the encoder and reconstructs the statistics of the polyp disease. In the decoder, the five up-sampling blocks containing 64, 48, 32, 16, and 8 filters are created. Each block contains many layers, including the convolution layer, batch normalization (BN), activation layer, up-sampling layer, and concatenation layer (CNC), which are used for synthesizing the information using a skip connection. A layer detail summary of the complete model is shown in Table 2. The notation used in Table 2 is defined as A—activation, C—convolution layer, BN—batch normalization, UP—up-sampling, MP—max pooling, CNC—concatenation layer.

In each phase of the USB, the convolution operation is performed and features from the feature maps are normalized by the BN layer. The activation function is applied on the normalized feature maps. Similarly, the information of each frame is forwarded to the next USB up to the last convolution layer, as shown in Figure 3. The dimensionality of the feature maps is kept the same across layers in the decoder block for the addition of the feature map at each stage in terms of skip connection. The skip connection provides the hidden information, which is misplaced due to the deepness of the encoder block network. It assists in better reconstruction of the semantic feature maps to the encoder, where the following residual block helps to learn the necessary features using backpropagation by repeating it many times. After the last convolution operation, the sigmoid activation function is performed, which provides the segmented frame as the final output of the Graft-U-Net model.

## 4. Results and Discussion

In this section, an explanation of the two datasets is given and performance evaluation protocols are addressed. A detailed description of the experiments is provided with the two datasets. The training and testing of the model were performed using the NVIDIA GTX 1070 GPU. Windows 10 was used with a core i5 machine with 8 GB of inbuilt RAM. Python Spyder IDE was used for the compiling of results and model evaluation. This section addresses the model’s performance evaluation protocols, visualizations of features, and experimental setup.

### 4.1. Datasets

**Figure 5:**
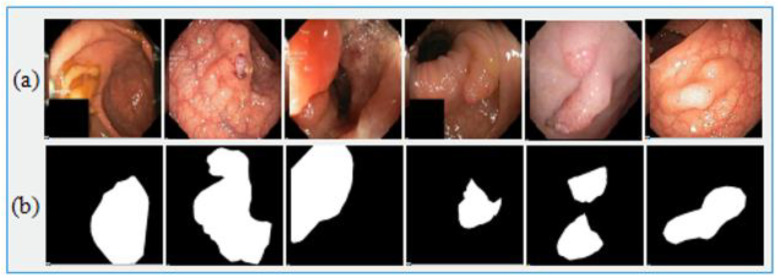
Sample Kvasir-SEG dataset images. (**a**) Original images and (**b**) ground truth images.

Medical-image analysis is a highly demanding task whereby pixel-wise image segmentation is performed using medical-imaging datasets. An open-access dataset such as Kvasir-SEG is an annotated medical-image dataset with a corresponding segmentation mask. The size of the file containing polyp frames was 46.2 MB. The original frames and their corresponding ground truth frames were verified by a qualified gastroenterologist. The resolution of the frames varied from 332 × 487 to 1920 × 1072 pixels in the whole dataset, which was stored in two folders—the actual images folder and the ground truth images folder—where the name of each frame was kept the same as the name of the original images in folder 1. The Kvasir-SEG dataset [58] was used for the evaluation of Graft-U-Net, which consisted of 1000 polyp images. The dataset was prepared by an expert endoscopist from Oslo University Hospital Norway (OUHN). An open-access CVC-ClinicDB dataset was employed as the state-of-the-art, and had 612 images with a 384 × 288 resolution from 31 colonoscopy sequences [59]. CVC-ClinicDB was also composed of two folders—one folder for original images and the other for ground truth images (containing a mask) corresponding to the polyp area covered in the original frame. A comprehensive summary of both datasets that are used in the proposed model is given in Table 3. Figure 5 illustrates the sample of the original images with corresponding ground truth images.

### 4.2. Performance Evaluation Measures

The standard computer vision methods for semantic segmentation were used for the evaluation of model performance using the Kvasir-SEG dataset in terms of precision, mean Dice coefficient (mDice), recall, accuracy, mean intersection of union (IoU), and F2-score. Each evaluation protocol provides specific information relevant to the experiment. A false-positive (fp) determines the information about a predicted class as positive when it is actually found to be negative, a true-positive (tp) provides a correct prediction, a false-negative (fn) considers the predicted class as negative while it is actually positive, and for a true-negative (tn), the actual class and predicted class are found to be negative. Test score accuracy is measured by the F2 scores that are used in binary classification problems.

(10) $$\mathrm{Mean}\;\mathrm{Dice}=\frac{2\times tp}{2*tp+fp+fn}$$

(11) $$\mathrm{mIoU}=\frac{tp}{tp+fp+fn}$$

(12) $$\mathrm{Recall}=\frac{tp}{tp+fn}$$

(13) $$\mathrm{Precision}=\frac{tp}{tp+fp}$$

(14) $$F_{2}=\frac{5p\times r}{4p+r}$$

(15) $$\mathrm{Accuracy}=\frac{tp+tn}{tp+tn+fp+fn}$$

### 4.3. Experiment 1: Results of Kvasir-SEG Dataset Using Graft-U-Net

**Figure 6:**
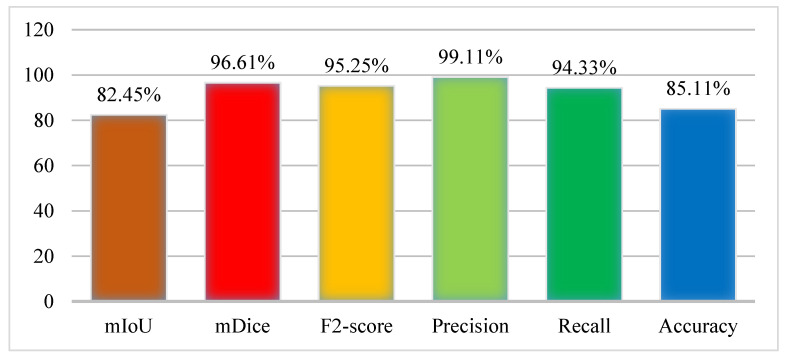
Results of Graft-U-Net with Kvasir-SEG dataset.

The experiment was performed using the Kvasir-SEG dataset where the ratio of the sample was set as 70% training and 30% testing. In the training, the number of epochs was set as 40 on 1000 frames of the dataset. The results were collected from the model in terms of performance evaluation metrics as the mIoU (82.45%), mDice (96.61%), F2 score (95.25%), Precision (99.11%), Recall (94.33%), and Accuracy (85.11%). The results of the Graft-U-Net model were obtained using the Kvasir-SEG dataset and are displayed in Figure 6, which makes the results of the model more noticeable.

**Figure 7:**
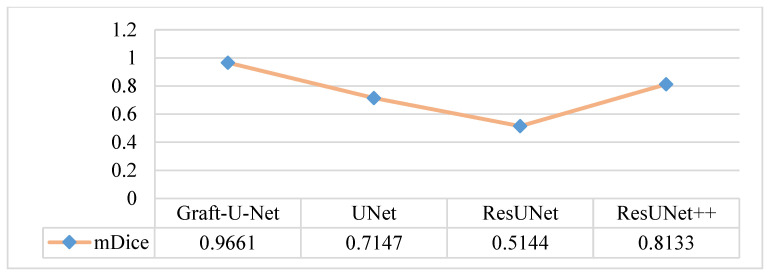
Comparison of Graft-U-Net on Kvasir-SEG dataset in terms of mDice.

The results of Graft-U-Net (0.9661) in terms of mDice are compared with the pre-study models of UNet (0.7147), ResUNet (0.5144), and ResUNet++ (0.8133). The analysis declares that the suggested model performs better in terms of mDice when using the Kvasir-SEG dataset. The Graft-U-Net architecture performs marginally well against the baseline architecture. The outcome of mDice on the Kvasir-SEG dataset is shown in Figure 7.

**Figure 8:**
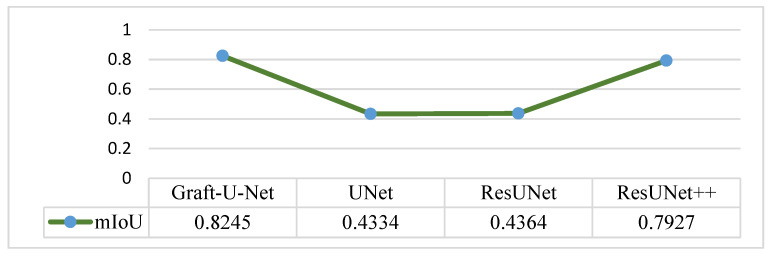
Comparison of Graft-U-Net on Kvasir-SEG dataset in terms of mIoU.

The results of Graft-U-Net (0.8245) in terms of mIoU are compared with the pre-study models of UNet (0.4334), ResUNet (0.4364), and ResUNet++ (0.7927). The comparison result of mIoU is depicted in Figure 8.

**Figure 9:**
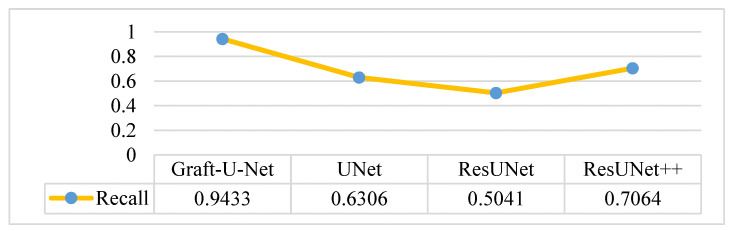
Comparison of Graft-U-Net on Kvasir-SEG dataset in terms of recall.

The results of Graft-U-Net (0.9433) in terms of Recall are compared with the pre-study models of UNet (0.6306), ResUNet (0.5041), and ResUNet++ (0.7064). The outcome shows that recall is enhanced when using the Kvasir-SEG dataset. The outcome of the recall is illustrated in the form of a table and a graph in Figure 9.

**Figure 10:**
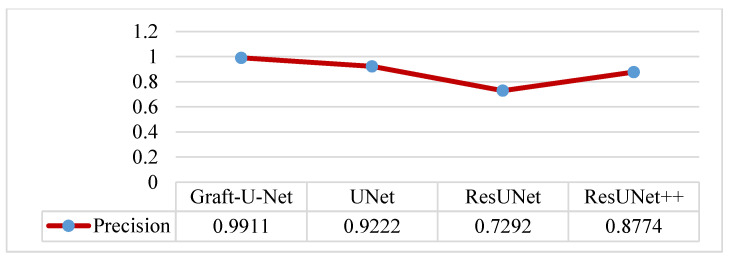
Comparison of Graft-U-Net on Kvasir-SEG dataset in terms of precision.

The results of Graft-U-Net (0.9911) in terms of precision are compared with the pre-study models of UNet (0.9222), ResUNet (0.7292), and ResUNet++ (0.8774). The result of the precision is improved. The combined form of the Precision results, including a table and a graph, are presented in Figure 10.

**Figure 11:**
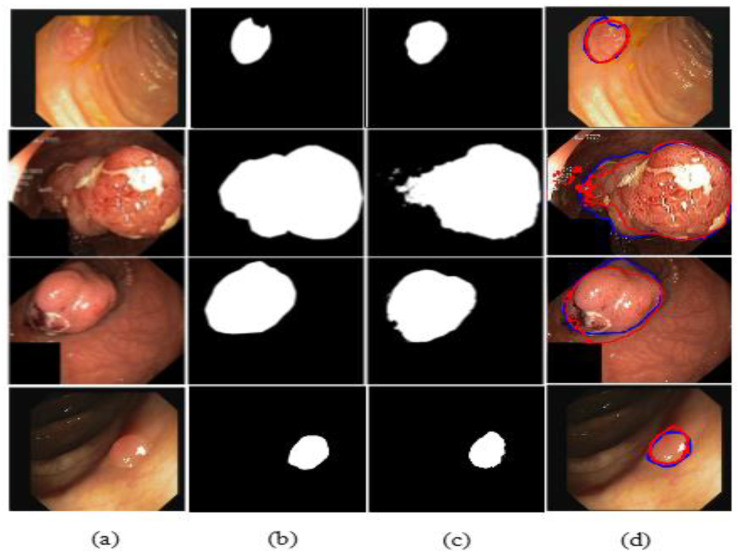
Comparison of ground truth and output mask on the Kvasir-SEG dataset. (**a**) Original Image; (**b**) ground truth; (**c**) output mask; and (**d**) combined.

The illustrated results are compiled on the Kvasir-SEG dataset and input images (original images) with the corresponding ground truth masks, model-predicted output masks, and a combined form of the ground truth masks and predicted masks, which are compared with a blue outline and a red outline, respectively, in Figure 11.

### 4.4. Experiment 2: Results of the CVC-ClinicDB Dataset Using Graft-U-Net

**Figure 12:**
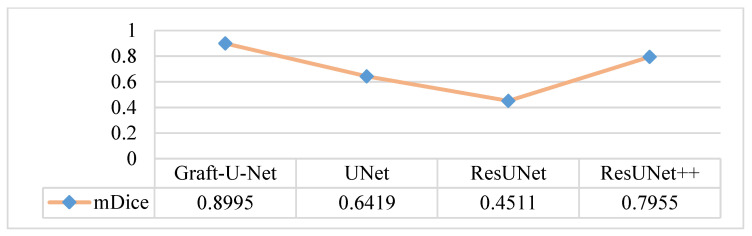
Comparison of Graft-U-Net on CVC-ClinicDB Dataset in terms of mDice.

An in-depth performance analysis and additional experiments were performed for automatic polyp segmentation. The CVC-ClinicDB dataset is considered the one that can make the model clinically acceptable. The results of Graft-U-Net (0.8995) in terms of mDice are compared with the pre-study models of UNet (0.6419), ResUNet (0.4511), and ResUNet++ (0.7955). The analysis declares that the proposed model provides improved results in terms of mDice when using the CVC-ClinicDB dataset. Figure 12 represents the result of the mDice.

**Figure 13:**
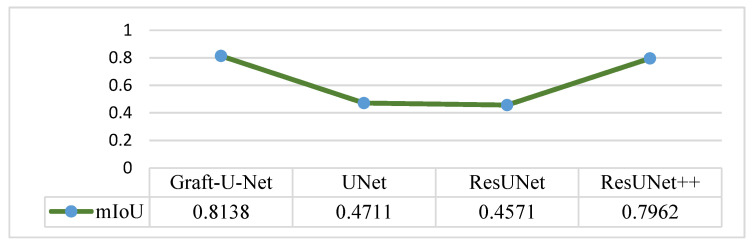
Comparison of Graft-U-Net on CVC-ClinicDB Dataset in terms of mIoU.

The results of Graft-U-Net (0.8138) in terms of mIoU are compared with the pre-study models of UNet (0.4711), ResUNet (0.4571), and ResUNet++ (0.7962). The comparison that is shown in Figure 13 declares the model to be better in terms of mIoU when using the CVC-ClinicDB dataset.

**Figure 14:**
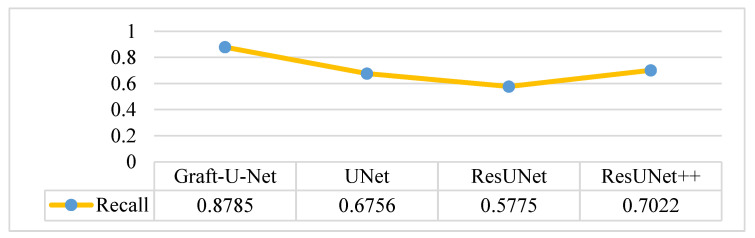
Comparison of Graft-U-Net on CVC-ClinicDB Dataset in terms of Recall.

The results of Graft-U-Net (0.8785) in terms of Recall are shown in Figure 14. The results are compared with the pre-study models of UNet (0.6756), ResUNet (0.5775), and ResUNet++ (0.7022) and are provided in the form of a table and a graph. The recall is improved using the CVC-ClinicDB dataset.

**Figure 15:**
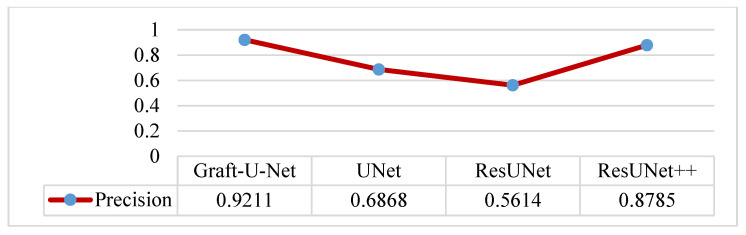
Comparison of Graft-U-Net on CVC-ClinicDB Dataset in terms of precision.

The results of Graft-U-Net (0.9211) in terms of Precision are compared with the existing work of UNet (0.6868), ResUNet (0.5614), and ResUNet++ (0.8785). The model outperforms in terms of precision when using the CVC-ClinicDB dataset. The integrated results, including a table and a graph, are presented in Figure 15.

**Figure 16:**
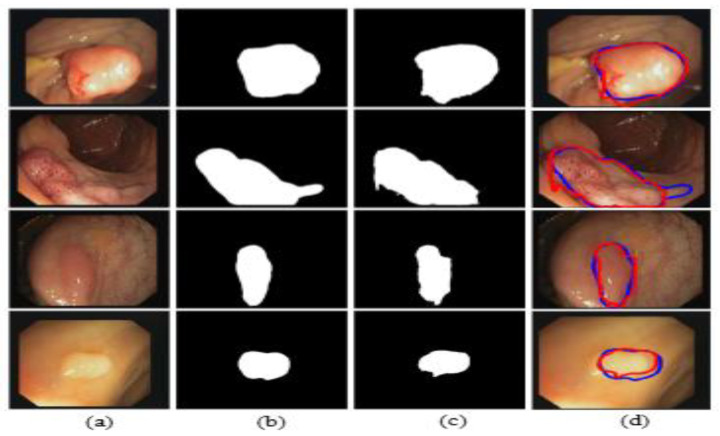
Comparison of ground truth and output mask on the CVC-ClinicDB dataset. (**a**) Original Image; (**b**) ground truth; (**c**) output mask; and (**d**) combined.

The illustrated results are compiled on the CVC-ClinicDB dataset and the input images (original images) with the corresponding ground truth masks, model-predicted output masks, and a combined form of the ground truth mask and predicted masks, which are compared with a blue outline and a red outline, respectively, in Figure 16.

### 4.5. Discussion

Semantic segmentation is a crucial segmentation technique that is employed for polyp detection from the frames of the GI tract. Deep learning plays a decisive role in the computer vision field for feature learning using CNN techniques. The challenges mostly occur in data acquisition, such as the appearance of the polyps fluctuating under the same lighting conditions and variable texture, and varying angular views under different lighting conditions. Graft-U-Net is the proposed method for polyp segmentation in our manuscript, and overcomes the addressed challenges attractively. The proposed model comprises main two blocks (encoder and decoder) where a graft network is proposed in the encoder block, as shown in Figure 3. The encoder analyzes the information in the frames while the decoder synthesizes the visual information using skip connections. Graft-U-Net outperforms in terms of mDice (96.61%), the mIoU (82.45%), precision (94.33%), and recall (99.11%) using the Kvasir-SEG dataset. Similarly, model performance was analyzed using the CVC-ClinicDB dataset, which provides better results in terms of mDice (89.95%), the mIoU (81.38%), precision (87.85%), and recall (92.11). Consequently, the algorithm can be made more generalized by using small-sized polyps for semantic segmentation. In this regard, Graft-U-Net is proposed to handle small polyps and shape information, and to incorporate artifacts separately to improve the model’s overall efficiency.

## 5. Conclusions and Future Work

The proposed Graft-U-Net model performs semantic segmentation better than existing models. The model achieves accurate segmentation of colorectal polyps using the two polyp datasets described in the manuscript. During the preprocessing phase, the CLAHE technique is used to enhance the intensity level of the frames of the Kvasir-SEG dataset. The proposed Graft-U-Net model is composed of encoder and decoder blocks where five DSBs and five USBs are made. The graft network is proposed in each DSB block in the encoder to obtain better feature maps. The decoder block constructs the feature maps for finding the location of the mask, which is the area covered in the original frame. So, the proposed model outperforms with respect to mDice (96.61%), the mIoU (82.45%), precision (94.33%), and recall (99.11%) using the Kvasir-SEG dataset; similarly, on the CVC-clinicDB dataset, the model achieves better results with mDice (89.95%), the mIoU (81.38%), precision (87.85%), and recall (92.11%). The performance evaluations are compared with the existing state-of-the-art models UNet, ResUNet, and ResUNet++.

The encoder block can be replaced by models including resNet, VGG, InceptionNet, AlexNet, etc. The proposed model can serve as a strong baseline for additional exploration to establish a useful technique, which will help to achieve the generalizability goal.

## Figures and Tables

**Table 1:** A review of the existing models employed for polyp segmentation on the Kvasir-SEG dataset.

Refs.	Years	Type of CNN	Dataset	Results (mDice)
[47]	2022	AMNet	Kvasir-SEG	91.20%
[48]	2022	BSCA-Net		91.00%
[49]	2022	SwinE-Net		93.80%
[50]	2021	MSNet		90.70%
[51]	2021	SANet		90.40%
[52]	2021	UACANet		90.50%

**Table 2:** Detailed layer information on the Graft-U-Net Architecture.

Layer No	Network Layers	Feature Map Dimension	Sliding Window Size	Stride Information	Padding Size	Pooling Window Details
1	Input	512 × 512 × 3	3 × 3 × 3 × 8	[1 1]	[0 0 0 0]	-
2,3,4	C1, BN1,A1	512 × 512 × 8	3 × 3 × 3 × 8	[1 1]	Same	-
5,6,7	C2,BN2,A2	512 × 512 × 8	3 × 3 × 3 × 8	[1 1]	Same	-
8,9,10	C3,BN3,A3	512 × 512 × 8	3 × 3 × 3 × 8	[1 1]	Same	-
11	MP	256 × 256 × 8	3 × 3 × 3 × 8	[1 1]	Same	Max pooling 3 × 3
12,13,14	C4,BN4,A4	256 × 256 × 16	3 × 3 × 3 × 16	[1 1]	Same	-
15,16,17	C5,BN5,A5	256 × 256 × 16	3 × 3 × 3 × 16	[1 1]	Same	-
18,19,20	C6,BN6,A6	256 × 256 × 16	3 × 3 × 3 × 16	[1 1]	Same	-
21	MP	128 × 128 × 16	3 × 3 × 3 × 16	[1 1]	Same	Max pooling 3 × 3
22,23,24	C7,BN7,A7	128 × 128 × 32	3 × 3 × 3 × 32	[1 1]	Same	-
25,26,27	C8,BN8,A8	128 × 128 × 32	3 × 3 × 3 × 32	[1 1]	Same	-
28,29,30	C9,BN9,A9	128 × 128 × 32	3 × 3 × 3 × 32	[1 1]	Same	-
31	MP	64 × 64 × 32	3 × 3 × 3 × 32	[1 1]	Same	Max pooling 3 × 3
32,33,34	C10,BN10,A10	64 × 64 × 48	3 × 3 × 3 × 48	[1 1]	Same	-
35,36,37	C11,BN11,A11	64 × 64 × 48	3 × 3 × 3 × 48	[1 1]	Same	-
38,39,40	C12,BN11,A12	64 × 64 × 48	3 × 3 × 3 × 48	[1 1]	Same	-
41	MP	32 × 32 × 48	3 × 3 × 3 × 48	[1 1]	Same	Max pooling 3 × 3
42,43,44	C13,BN13,A13	32 × 32 × 64	3 × 3 × 3 × 64	[1 1]	Same	-
45,46,47	C14,BN14,A14	32 × 32 × 64	3 × 3 × 3 × 64	[1 1]	Same	-
48,49,50	C15,BN15,A15	32 × 32 × 64	3 × 3 × 3 × 64	[1 1]	Same	-
51	MP	16 × 16 × 64	3 × 3 × 3 × 64	[1 1]	Same	Max pooling 3 × 3
52,53,54	C16,BN16,A16	16 × 16 × 64	3 × 3 × 3 × 64	[1 1]	Same	-
55	UPS1	32 × 32 × 64	3 × 3 × 3 × 64	[1 1]	Same	-
56	CNC1	32 × 32 × 128	-	-	-	-
57,58,59	C17,BN17,A17	32 × 32 × 64	3 × 3 × 3 × 64	[1 1]	Same	-
60	UPS2	64 × 64 × 64	3 × 3 × 3 × 64	[1 1]	Same	-
61	CNC2	64 × 64 × 112	-	-	-	-
62,63,64	C18,BN18,A18	64 × 64 × 48	3 × 3 × 3 × 48	[1 1]	Same	-
65	UPS3	128 × 128 × 48	3 × 3 × 3 × 48	[1 1]	Same	-
66	CNC3	128 × 128 × 80	-	-	-	-
67,68,69	C19,BN19,A19	128 × 128 × 32	3 × 3 × 3 × 32	[1 1]	Same	-
70	UPS4	256 × 256 × 32	3 × 3 × 3 × 32	[1 1]	Same	-
71	CNC4	256 × 256 × 48	-	-	-	-
72,73,74	C20,BN20,A20	256 × 256 × 16	3 × 3 × 3 × 16	[1 1]	Same	-
75	UPS5	512 × 512 × 16	3 × 3 × 3 × 16	[1 1]	Same	-
76	CNC5	512 × 512 × 24	-	-	-	-
77,78,79	C21,BN21,A21	512 × 512 × 8	3 × 3 × 3 × 8	[1 1]	Same	-
80,81	C22,A22	512 × 512 × 1	3 × 3 × 3 × 1	[1 1]	Same	-

**Table 3:** Summary of the Datasets.

Datasets	Organs	Diseases	Modalities	Original Images	Ground Truth
CVC-ClinicDB [60]	Bowel Colon	Polyps	Colonoscopy	612 images	612 images
Kvasir-SEG [58]	Bowel Colon	Polyps	Colonoscopy	1000 images	1000 images

## Data Availability

Not applicable.

## References

[1] Grady W.M. (2021). Epigenetic Alterations in the Gastrointestinal Tract: Current and Emerging Use for Biomarkers of Cancer. Adv. Cancer Res..

[2] Naz J., Sharif M., Raza M., Shah J.H., Yasmin M., Kadry S., Vimal S. (2021). Recognizing Gastrointestinal Malignancies on WCE and CCE Images by an Ensemble of Deep and Handcrafted Features with Entropy and PCA Based Features Optimization. Neural Process. Lett..

[3] Nagahara A., Shiotani A., Iijima K., Kamada T., Fujiwara Y., Kasugai K., Kato M., Higuchi K. (2021). The Role of Advanced Endoscopy in The Management of Inflammatory Digestive Diseases (upper gastrointestinal tract). Dig. Endosc..

[4] Naz J., Sharif M., Yasmin M., Raza M., Khan M.A. (2021). Detection and Classification of Gastrointestinal Diseases using Machine Learning. Curr. Med. Imaging Former. Curr. Med Imaging Rev..

[5] Chen P.-J., Lin M.-C., Lai M.-J., Lin J.-C., Lu H.H.-S., Tseng V.S. (2018). Accurate Classification of Diminutive Colorectal Polyps Using Computer-Aided Analysis. Gastroenterology.

[6] Banik D., Roy K., Bhattacharjee D., Nasipuri M., Krejcar O. (2020). Polyp-Net: A Multimodel Fusion Network for Polyp Segmentation. IEEE Trans. Instrum. Meas..

[7] Sun S., Guo J., Bhutani M.S., Giovannini M., Li Z., Jin Z., Yang A., Xu G., Wang G. (2017). Can Endoscopic Ultrasound-Guided Needle-Based Confocal Laser Endomicroscopy Replace Fine-Needle Aspiration for Pancreatic and Mediastinal Diseases?. Endosc. Ultrasound.

[8] Yang Y.J., Cho B.-J., Lee M.-J., Kim J.H., Lim H., Bang C.S., Jeong H.M., Hong J.T., Baik G.H. (2020). Automated Classification of Colorectal Neoplasms in White-Light Colonoscopy Images via Deep Learning. J. Clin. Med..

[9] Mori Y., Kudo S., Ikehara N., Wakamura K., Wada Y., Kutsukawa M., Misawa M., Kudo T., Kobayashi Y., Miyachi H. (2013). Comprehensive Diagnostic Ability of Endocytoscopy Compared with Biopsy for Colorectal Neoplasms: A Prospective Randomized Noninferiority Trial. Laryngo-Rhino-Otologie.

[10] Tomar N.K. (2021). Automatic Polyp Segmentation using Fully Convolutional Neural Network. arXiv.

[11] Kronborg O., Regula J. (2007). Population Screening for Colorectal Cancer: Advantages and Drawbacks. Dig. Dis..

[12] Riegler M. (2017). EIR-A Medical Multimedia System for Efficient Computer Aided Diagnosis. Ph.D. Thesis.

[13] Ramzan M., Raza M., Sharif M., Khan M.A., Nam Y. (2021). Gastrointestinal Tract Infections Classification Using Deep Learning. Comput. Mater. Contin..

[14] Rasheed S., Raza M., Sharif M., Kadry S., Alharbi A. (2022). Single Channel Image Enhancement (SCIE) of White Blood Cells Based on Virtual Hexagonal Filter (VHF) Designed over Square Trellis. J. Pers. Med..

[15] Amin J., Anjum M.A., Sharif A., Raza M., Kadry S., Nam Y. (2022). Malaria Parasite Detection Using a Quantum-Convolutional Network. Cmc-Comput. Mater. Contin..

[16] Shahzad A., Raza M., Shah J.H., Sharif M., Nayak R.S. (2022). Categorizing White Blood Cells by Utilizing Deep Features of Proposed 4B-Additionnet-Based CNN Network with Ant Colony Optimization. Complex Intell. Syst..

[17] Naz M., Shah J.H., Khan M.A., Sharif M., Raza M., Damaševičius R. (2021). From ECG Signals to Images: A Transformation Based Approach for Deep Learning. PeerJ Comput. Sci..

[18] Amin J., Sharif M., Anjum M.A., Siddiqa A., Kadry S., Nam Y., Raza M. (2021). 3D Semantic Deep Learning Networks for Leukemia Detection. Comput. Mater. Contin..

[19] Sharif M.I., Khan M.A., Alhussein M., Aurangzeb K., Raza M. (2022). A decision support system for multimodal brain tumor classification using deep learning. Complex Intell. Syst..

[20] Shin Y., Qadir H.A., Balasingham I. (2018). Abnormal Colon Polyp Image Synthesis Using Conditional Adversarial Networks for Improved Detection Performance. IEEE Access.

[21] Huang C.-H., Wu H.-Y., Lin Y.-L. (2021). HarDNet-MSEG: A Simple Encoder-Decoder Polyp Segmentation Neural Network that Achieves over 0.9 Mean Dice and 86 FPS. arXiv.

[22] Nogueira-Rodríguez A., Domínguez-Carbajales R., López-Fernández H., Iglesias J., Cubiella J., Fdez-Riverola F., Reboiro-Jato M., Glez-Peña D. (2020). Deep Neural Networks approaches for detecting and classifying colorectal polyps. Neurocomputing.

[23] Liu W.N., Zhang Y.Y., Bian X.Q., Wang L.J., Yang Q., Zhang X.D., Huang J. (2020). Study on Detection Rate of Polyps and Adenomas in Artificial-Intelligence-Aided Colonoscopy. Saudi J. Gastroenterol. Off. J. Saudi Gastroenterol. Assoc..

[24] Lee J.Y., Jeong J., Song E.M., Ha C., Lee H.J., Koo J.E., Yang D.-H., Kim N., Byeon J.-S. (2020). Real-Time Detection of Colon Polyps During Colonoscopy Using Deep Learning: Systematic Validation with Four Independent Datasets. Sci. Rep..

[25] Ronneberger O., Fischer P., Brox T. (2015). U-net: Convolutional Networks for Biomedical Image Segmentation. International Conference on Medical Image Computing and Computer-Assisted Intervention.

[26] Zhang Z., Liu Q., Wang Y. (2018). Road Extraction by Deep Residual U-Net. IEEE Geosci. Remote Sens. Lett..

[27] Jha D., Smedsrud P.H., Riegler M.A., Johansen D., De Lange T., Halvorsen P., Johansen H.D. Resunet++: An Advanced Architecture for Medical Image Segmentation. Proceedings of the 2019 IEEE International Symposium on Multimedia (ISM).

[28] Sharif M., Amin J., Raza M., Anjum M.A., Afzal H., Shad S.A. (2020). Brain Tumor Detection Based on Extreme Learning. Neural Comput. Appl..

[29] Amin J., Sharif M., Raza M., Saba T., Sial R., Shad S.A. (2019). Brain Tumor Detection: A Long Short-Term Memory (LSTM)-Based Learning Model. Neural Comput. Appl..

[30] Amin J., Sharif M., Gul N., Raza M., Anjum M.A., Nisar M.W., Bukhari S.A.C. (2020). Brain Tumor Detection by Using Stacked Autoencoders in Deep Learning. J. Med. Syst..

[31] Amin J., Sharif M., Raza M., Saba T., Anjum M.A. (2019). Brain Tumor Detection Using Statistical and Machine Learning Method. Comput. Methods Programs Biomed..

[32] Amin J., Sharif M., Raza M., Saba T., Rehman A. Brain Tumor Classification: Feature Fusion. Proceedings of the 2019 International Conference on Computer and Information Sciences (ICCIS).

[33] Khan M.A., Sharif M.I., Raza M., Anjum A., Saba T., Shad S.A. (2019). Skin Lesion Segmentation and Classification: A Unified Framework of Deep Neural Network Features Fusion and Selection. Expert Syst..

[34] Saba T., Bokhari S.T.F., Sharif M., Yasmin M., Raza M. (2018). Fundus Image Classification Methods for the Detection of Glaucoma: A Review. Microsc. Res. Technol..

[35] Amin J., Sharif M., Rehman A., Raza M., Mufti M.R. (2018). Diabetic Retinopathy Detection and Classification Using Hybrid Feature Set. Microsc. Res. Technol..

[36] Ameling S., Wirth S., Paulus D., Lacey G., Vilarino F. (2009). Texture-Based Polyp Detection in Colonoscopy. Bildverarbeitung für die Medizin 2009.

[37] Bernal J., Tajkbaksh N., Sanchez F.J., Matuszewski B.J., Chen H., Yu L., Angermann Q., Romain O., Rustad B., Balasingham I. (2017). Comparative Validation of Polyp Detection Methods in Video Colonoscopy: Results from the MICCAI 2015 Endoscopic Vision Challenge. IEEE Trans. Med. Imaging.

[38] Wang Y., Tavanapong W., Wong J., Oh J.H., De Groen P.C. (2015). Polyp-Alert: Near Real-Time Feedback During Colonoscopy. Comput. Methods Programs Biomed..

[39] Shin Y., Qadir H.A., Aabakken L., Bergsland J., Balasingham I. (2018). Automatic Colon Polyp Detection Using Region Based Deep CNN and Post Learning Approaches. IEEE Access.

[40] Goodfellow I.J., Pouget-Abadie J., Mirza M., Xu B., Warde-Farley D., Ozair S., Courville A., Bengio Y. (2014). Generative Adversarial Networks. arXiv.

[41] Redmon J., Divvala S., Girshick R., Farhadi A. You Only Look Once: Unified, Real-Time Object Detection. Proceedings of the 2016 IEEE Conference on Computer Vision and Pattern Recognition (CVPR).

[42] Yamada M., Saito Y., Imaoka H., Saiko M., Yamada S., Kondo H., Takamaru H., Sakamoto T., Sese J., Kuchiba A. (2019). Development of a Real-Time Endoscopic Image Diagnosis Support System Using Deep Learning Technology in Colonoscopy. Sci. Rep..

[43] Esteva A., Chou K., Yeung S., Naik N., Madani A., Mottaghi A., Liu Y., Topol E., Dean J., Socher R. (2021). Deep Learning-Enabled Medical Computer Vision. NPJ Digit. Med..

[44] Yeung M., Sala E., Schönlieb C.-B., Rundo L. (2021). Unified Focal loss: Generalising Dice and Cross Entropy-Based Losses to Handle Class Imbalanced Medical Image Segmentation. Comput. Med. Imaging Graph..

[45] Long J., Shelhamer E., Darrell T. Fully Convolutional Networks for Semantic Segmentation. Proceedings of the IEEE Conference on Computer Vision and Pattern Recognition (CVPR).

[46] Amin J., Sharif M., Anjum M.A., Raza M., Bukhari S.A.C. (2020). Convolutional Neural Network with Batch Normalization for Glioma and Stroke Lesion Detection Using MRI. Cogn. Syst. Res..

[47] Song P., Li J., Fan H. (2022). Attention Based Multi-Scale Parallel Network for Polyp Segmentation. Comput. Biol. Med..

[48] Lin Y., Wu J., Xiao G., Guo J., Chen G., Ma J. (2022). BSCA-Net: Bit Slicing Context Attention Network for Polyp Segmentation. Pattern Recognit..

[49] Park K.B., Lee J.Y. (2022). SwinE-Net: Hybrid Deep Learning Approach to Novel Polyp Segmentation Using Convolutional Neural Network and Swin Transformer. J. Comput. Des. Eng..

[50] Zhao X., Zhang L., Lu H. Automatic Polyp Segmentation Via Multi-Scale Subtraction Network. Proceedings of the International Conference on Medical Image Computing and Computer-Assisted Intervention.

[51] Wei J., Hu Y., Zhang R., Li Z., Zhou S.K., Cui S. Shallow Attention Network for Polyp Segmentation. Proceedings of the International Conference on Medical Image Computing and Computer-Assisted Intervention.

[52] Kim T., Lee H., Kim D. Uacanet: Uncertainty Augmented Context Attention for Polyp Segmentation. Proceedings of the 29th ACM International Conference on Multimedia.

[53] Hasan M., Islam N., Rahman M.M. (2020). Gastrointestinal Polyp Detection Through a Fusion of Contourlet Transform and Neural Features. J. King Saud Univ.-Comput. Inf. Sci..

[54] Kang J., Gwak J. (2019). Ensemble of Instance Segmentation Models for Polyp Segmentation in Colonoscopy Images. IEEE Access.

[55] Kassani S.H., Kassani P.H., Wesolowski M.J., Schneider K.A., Deters R. (2021). Deep Transfer Learning Based Model for Colorectal Cancer Histopathology Segmentation: A Comparative Study of Deep Pre-Trained Models. Int. J. Med. Informatics.

[56] Ribeiro J., Nóbrega S., Cunha A. (2022). Polyps Detection in Colonoscopies. Procedia Comput. Sci..

[57] Ioffe S., Szegedy C. (2015). Batch Normalization: Accelerating Deep Network Training by Reducing Internal Covariate Shift. Proceedings of the International Conference on Machine Learning.

[58] Jha D., Smedsrud P.H., Riegler M.A., Halvorsen P., Lange T.D., Johansen D., Johansen H.D. Kvasir-Seg: A Segmented Polyp Dataset. Proceedings of the International Conference on Multimedia Modeling.

[59] Bernal J., Sánchez F.J., Fernández-Esparrach G., Gil D., Rodríguez C., Vilariño F. (2015). WM-DOVA Maps for Accurate Polyp Highlighting in Colonoscopy: Validation Vs. Saliency Maps from Physicians. Comput. Med. Imaging Graph..

[60] Jha D., Riegler M.A., Johansen D., Halvorsen P., Johansen H.D. Doubleu-Net: A Deep Convolutional Neural Network for Medical Image Segmentation. Proceedings of the 2020 IEEE 33rd International Symposium on Computer-Based Medical Systems (CBMS).

